# Proposed Diagnostic Criteria for Smartphone Addiction

**DOI:** 10.1371/journal.pone.0163010

**Published:** 2016-11-15

**Authors:** Yu-Hsuan Lin, Chih-Lin Chiang, Po-Hsien Lin, Li-Ren Chang, Chih-Hung Ko, Yang-Han Lee, Sheng-Hsuan Lin

**Affiliations:** 1 Department of Psychiatry, National Taiwan University Hospital, Taipei, Taiwan; 2 Department of Psychiatry, Shin Kong Wu Ho-Su Memorial Hospital, Taipei, Taiwan; 3 Division of New Drugs, Center for Drug Evaluation, Taiwan; 4 Department of Psychiatry, Koo Foundation Sun Yat-Sen Cancer Center, New Taipei City, Taiwan; 5 Department of Psychiatry, National Taiwan University, College of Medicine, Taipei, Taiwan; 6 Department of Psychiatry, Kaohsiung Medical University, Kaohsiung, Taiwan; 7 Department of Psychiatry, Kaohsiung Municipal Hsiao-Kang Hospital, Kaohsiung, Taiwan; 8 Department and Graduate School of Electrical Engineering, Tamkang University Hospital, New Taipei City, Taiwan; 9 Department of Biostatistics, Columbia Mailman School of Public Health, New York, New York, United States of America; Ariel University, ISRAEL

## Abstract

**Background:**

Global smartphone penetration has led to unprecedented addictive behaviors. The aims of this study are to develop diagnostic criteria of smartphone addiction and to examine the discriminative ability and the validity of the diagnostic criteria.

**Methods:**

We developed twelve candidate criteria for characteristic symptoms of smartphone addiction and four criteria for functional impairment caused by excessive smartphone use. The participants consisted of 281 college students. Each participant was systematically assessed for smartphone-using behaviors by psychiatrist’s structured diagnostic interview. The sensitivity, specificity, and diagnostic accuracy of the candidate symptom criteria were analyzed with reference to the psychiatrists’ clinical global impression. The optimal model selection with its cutoff point of the diagnostic criteria differentiating the smartphone addicted subjects from non-addicted subjects was then determined by the best diagnostic accuracy.

**Results:**

Six symptom criteria model with optimal cutoff point were determined based on the maximal diagnostic accuracy. The proposed smartphone addiction diagnostic criteria consisted of (1) six symptom criteria, (2) four functional impairment criteria and (3) exclusion criteria. Setting three symptom criteria as the cutoff point resulted in the highest diagnostic accuracy (84.3%), while the sensitivity and specificity were 79.4% and 87.5%, respectively. We suggested determining the functional impairment by two or more of the four domains considering the high accessibility and penetration of smartphone use.

**Conclusion:**

The diagnostic criteria of smartphone addiction demonstrated the core symptoms “impaired control” paralleled with substance related and addictive disorders. The functional impairment involved multiple domains provide a strict standard for clinical assessment.

## Introduction

Due to increasing smartphone penetration, excessive smartphone use and even smartphone addiction, one form of technological addictions, have become substantial worldwide social issues. “Smartphone addiction” is considered as one form of technological addictions. Griffiths [1] operationally defined technological addiction as one type of behavior addiction that involves human-machine interaction and is non-chemical in nature. The most well-known behavior addiction, gambling disorder, has been categorized to “substance related and addictive disorders” in the current version of the Diagnostic and Statistical Manual of Mental Disorders (DSM-5) because of the similar symptomatology, biological dysfunction [2], genetic liability [3], and treatment approach [4–6]. Another similar behavior pattern, Internet gaming disorder, has also been listed in the research criteria of DSM-5 [7]. Compared with computer use, the high accessibility of smartphone has led to overwhelming smartphone penetration, having attracted increasing attention on the investigation of smartphone addiction.

Several self-reported questionnaires were developed to assess the smartphone addiction over the recent years [8–14]. Generally, smartphone addiction consists of four main components: compulsive behaviors, tolerance, withdrawal, and functional impairment [12], which are identical to the components of Internet addiction [15]. In addition, two mobile applications (Apps) have been designed to identify smartphone addiction [12, 14, 16–18]. The App-generated parameters can delineate some symptoms of smartphone addiction, such as the excessive use and tolerance, and predict the diagnosis of smartphone addiction [14]. Both self-reported questionnaires and Apps are practical screening tools for large-scale epidemiologic studies. For health care professionals to confirm the diagnosis, our previous study proposed a set of diagnostic criteria for smartphone addiction, demonstrating that the smartphone addiction is similar to broad-spectrum Internet addiction rather than the specific Internet gaming disorder because smartphone use is characterized by the use of multiple Apps [14]. In previous study, 79 college students were recruited to examine twelve candidate symptom criteria and four functional impairment criteria. For accurately validating the diagnostic accuracy of the candidate criteria, a large scale study with more sample size is necessary.

The specific aim of this study is to validate the proposed criteria, including both the symptoms and functional impairment for smartphone addiction based on psychiatric interview. We hypothesized that smartphone addiction shared core symptoms with Internet addiction, but presented with unique features due to the high accessibility.

## Methods

### Participants

In total, 282 young adults were recruited from the Department of Electrical Engineering and of Computer and Communication Engineering of two universities in Northern Taiwan from December 2012 to June 2015. The recruitment strategy was based on the potential higher penetration rate of smartphone use among these students. In addition, male college students are the most high risk group in Internet addiction [19–21]. One participant with obsessive-compulsive disorder (OCD) was excluded from our study. Of these, 233 were male and 48 were female, with a mean age of 20.9 ± 1.6 years. All participants were provided written informed consent. The study was approved by the Institutional Review Board of National Taiwan University Hospital. The investigation was carried out in accordance with the latest version of the Declaration of Helsinki.

### Diagnostic criteria of smartphone addiction

We proposed a list of diagnostic criteria for smartphone addiction with three parts: the first part (criteria A) consisted of the symptoms of smartphone addiction and the second part (criteria B) described the functional impairment which was secondary to smartphone use. The final part was the exclusion criteria to rule out manic episodes and OCD (criterion C).

In criteria A, we developed twelve candidate diagnostic criteria to identify the characteristic symptoms of smartphone addiction based on the Diagnostic Criteria of Internet Addiction for College Students (candidate criterion A1-9) [22, 23] and on the research diagnostic criteria (Criterion A1, 3, 4, 6, 9–12) of Internet gaming disorder in DSM-5 (American Psychiatric Association, 2013). Four qualified psychiatrists, Lin YH, Lin PH, Chiang CL, and Chang LR, experienced in substance-related disorder and Internet addiction, modified these criteria for ‘‘smartphone addiction” assessment.

To evaluate the functional impairment, we summarized the description of functional impairment in the previous study [12] into three criteria (Criterion B-1, -2 and -3). Because smartphone addictive behavior is similar to compulsive behavior, we also applied “subjective distress or is time-consuming” from the criterion of OCD as the other aspect of functional impairment (Criterion B-4).

After interviewing every participant, all psychiatrists rated a clinical global impression (CGI) for the presence of smartphone addiction, according to their clinical experience and the concepts of addiction diagnosis proposed by West [24]. Our previous studies showed good inter-rater reliability of CGI with Fleiss-kappa up to 0.864 (which were rated by Lin YH, Lin PH, and Chang LR) [14] and 0.805 (by Lin YH, Lin PH, and Chiang CL) [25] respectively. Our previous studies also showed good test-retest reliability (agreement: 93.7%-100%) for each diagnostic criterion among the four psychiatrists (Lin YH, Lin PH, Chang LR, and Chiang CL) [25].

### The self-reported time spent on smartphone use

To assess the total duration of the participants' smartphone use, all participants were asked the average time for smartphone use during one weekday and the difference of the average time for smartphone use between weekday and weekend. If participants thought that their use pattern was too frequent to assess the total duration, the psychiatrists would help the participants to recall the majority of Apps they had used. If the participants were still unable to assess the smartphone use, they were coded as “frequent usage, very hard to estimate” and defined as “frequent users”.

### Statistical Analysis

The sensitivity, specificity, and diagnostic accuracy of the twelve candidate diagnostic criteria for smartphone addiction were evaluated between CGI-positive and CGI-negative groups. The diagnostic accuracy indicated the percentage of all correct decisions, which is the result of dividing the number of true positives and true negatives by the number of all decisions.

The candidate diagnostic criteria with lower diagnostic accuracy were excluded from further analyses. The cutoff point of the diagnostic criteria A differentiating the smartphone addicted subjects with non-addicted ones was then determined by the best diagnostic accuracy. The diagnostic criteria for smartphone addiction were constructed based on the cutoff point. We compared the maximal diagnostic accuracy among all models with different number of criteria (from four-criterion model to ten-criterion model) and chose one with highest diagnostic accuracy as our proposed diagnostic criteria.

Unlike the substance use, the smartphone use is not an illegally problematic behavior. Therefore, we adapted a strict definition of functional impairment (having two or more domains in criteria B). Participants who met both criteria A and B were diagnosed as smartphone addiction.

We compared the demographic data and characteristics between the smartphone addicted and non-addicted subjects by independent t-test for continuous variables and chi-square test for categorical variables. A *p*-value of less than 0.05 was considered statistically significant. All statistical analysis was conducted using SPSS v.18.0 (IBM Corp., Armonk, NY, USA).

## Results

Among 281 participants in the present study, as defined by the psychiatrists’ CGI ratings, 97 participants were classified as the CGI-positive group, while 184 participants were classified as the CGI-negative group. Using CGI as the provisional gold standard, the specificity, sensitivity, and diagnostic accuracy of the candidate diagnostic criteria for smartphone addiction were shown in Table 1. The diagnostic accuracy for twelve candidate symptom criteria was in the range of 67.6%-76.5%, while that for four functional impairment criteria in the range of 72.2%-79.7%.

**Table 1:** Profile of the candidate diagnostic criteria for smartphone addiction.

	Candidate Criteria	Proportion	Diagnostic accuracy	Sensitivity	Specificity	Positive Predictive Rate	Negative Predictive Rate
A1	Preoccupation with smartphone use, and hence keeping smartphone device available all day	29.9%	69.8%	49.5%	80.4%	57.1%	75.1%
A2	Recurrent failure to resist the impulse to use the smartphone	31.3%	74.7%	58.8%	83.2%	64.8%	79.3%
A3	Tolerance: a marked increase in the duration of smartphone use is needed to achieve satisfaction	27.1%	67.6%	42.3%	81.0%	53.9%	72.7%
A4	Withdrawal: manifested as a dysphoric mood, anxiety and irritability after a period without smartphone use	24.6%	70.1%	42.3%	84.8%	59.4%	73.6%
A5	Smartphone use for a period longer than intended	36.3%	71.2%	60.8%	76.6%	57.8%	78.8%
A6	Persistent desire and/or unsuccessful attempts to cut down or reduce smartphone use	24.2%	70.5%	42.3%	85.3%	60.3%	73.7%
A7	Excessive smartphone use and/or time spent on quitting the smartphone use	40.9%	76.5%	75.3%	77.2%	63.5%	85.5%
A8	Excessive effort spent on smartphone use as much as possible, even when it is inappropriate to use it	42.7%	68.3%	66.0%	69.6%	53.3%	79.5%
A9	Continued excessive smartphone use despite knowledge of having a persistent or recurrent physical or psychological problem resulting from smartphone overuse	30.5%	76.0%	59.4%	84.7%	67.1%	79.9%
A10	Use of the smartphone to escape or relieve a dysphoric mood (e.g. helpless, guilt, anxiety)	26.5%	69.9%	44.8%	83.1%	58.1%	74.2%
A11	Loss of previous interests, hobbies and entertainment as a result of—and with the exception of—smartphone use	11.8%	69.5%	22.9%	94.0%	66.7%	69.9%
A12	Deception of family members, therapists, or others regarding the amount of time spent on smartphone use	9.7%	69.5%	19.8%	95.6%	70.4%	69.4%
B1	Excessive smartphone use resulting in persistent or recurrent physical or psychological problems	27.4%	75.8%	54.6%	87.0%	68.8%	78.4%
B2	Smartphone use in situations in which it is physically hazardous (e.g., smartphone use while driving, or crossing the street) or significant negative impacts on daily life	28.1%	72.2%	50.5%	83.7%	62.0%	76.2%
B3	Smartphone use resulting in impairment of social relationships, schoolwork or job performance	30.6%	79.7%	64.9%	87.5%	73.3%	82.6%
B4	Excessive smartphone use causes significant subjective distress, or is time-consuming	31.3%	76.2%	60.8%	84.2%	67.1%	80.3%

Table 2 demonstrated the process to determine the optimal cutoff points in the six-criterion model. The six-criterion model consisted of six candidate criteria (A2, A4, A5, A6, A7, and A9) with higher diagnostic accuracies in the range of 70.1%-76.5%. The candidate criteria with lower diagnostic accuracies (A1, A3, A8, A10, A11, and A12) in the range of 67.6%-69.9% were excluded. The area under ROC curve (AUC) is 93.4%. The optimal cutoff point is at three, i.e. an individual with three positive criteria or more among A2, A4, A5, A6, A7, and A9, is included in the smartphone addiction group. With this optimal cutoff point, the six-criterion model performed the maximal diagnostic accuracy (84.3%), while the sensitivity and specificity are 79.4% and 87.5%, respectively.

**Table 2:** Cutoff point for criteria A within the six-diagnostic criteria model for smartphone addiction.

Cutoff Point	Diagnostic Accuracy	Sensitivity	Specificity	Positive Predictive Rate	Negative Predictive Rate
1	60.5%	99.0%	40.2%	46.6%	98.7%
2	77.9%	94.9%	69.0%	61.7%	96.2%
3	84.3%	78.4%	87.5%	76.8%	88.5%
4	77.9%	44.3%	95.7%	84.3%	76.5%
5	71.5%	18.6%	99.5%	94.7%	69.9%
6	66.5%	3.1%	100%	100%	66.2%

A cutoff point at three criteria of the six criteria model (A2, A4, A5, A6, A7, A9) performed the optimal diagnostic accuracy (84.3%).

Table 3 showed each model (from four-criterion model to ten-criterion model) with its optimal diagnostic accuracy. Similarly, the four-criterion model consisted of four candidate criteria (A2, A5, A7, and A9) with higher diagnostic accuracies in the range of 71.2%-76.5%. The candidate criteria (A1, A3, A4, A6, A8, A10, A11, and A12) with lower diagnostic accuracies in the range of 67.6%-70.5%, were excluded. The ten-criterion model consisted of ten candidate criteria (A1, A2, A4, A5, A6, A7, A9, A10, A11, and A12) with higher diagnostic accuracies in the range of 69.5%-76.5%. The candidate criteria (A3 and A8) with lower diagnostic accuracies, 67.6% and 68.3%, were excluded. There was a maximal diagnostic accuracy in the six-criterion model among these models.

**Table 3:** Select the model with the optimal cutoff points for with the maximal diagnostic accuracy.

Model	Criteria	Optimal Cutoff Points	Diagnostic Accuracy	Sensitivity	Specificity
4 criteria	A2, A5, A7, A9	3	81.1%	84.4%	80.2%
5 criteria	A2, A5, A6, A7, A9	3	82.9%	80.2%	84.0%
6 criteria	A2, A4, A5, A6, A7, A9	3	84.3%	78.4%	87.5%
7 criteria	A2, A4, A5, A6, A7, A9, A10	3	83.3%	72.3%	90.5%
7 criteria	A2, A4, A5, A6, A7, A9, A10	4	83.3%	86.8%	82.2%
8 criteria	A1, A2, A4, A5, A6, A7, A9, A10	4	82.9%	78.8%	84.7%
10 criteria	A1, A2, A4, A5, A6, A7, A9, A10, A11, A12	4	82.9%	78.8%	84.7%

Our final version of the diagnostic criteria was listed in Table 4. Criterion A consisted of the six characteristic symptom criteria and criterion B described the four functional impairment criteria which was secondary to smartphone use. According to the proposed diagnostic criteria for smartphone addiction, 65 participants were diagnosed as having smartphone addiction (the addicted group), and 216 were free from smartphone addiction (the non-addicted group).

**Table 4:** Proposed diagnostic criteria for smartphone addiction.

Criteria category	Description
**Criteria A**	Maladaptive pattern of smartphone use, leading to clinically significant impairment or distress, occurring at any time within the same 3-month period. Three (or more) of the following symptoms having been present:
	Recurrent failure to resist the impulse to use the smartphone Withdrawal: as manifested by dysphoria, anxiety and/or irritability after a period without smartphone use Smartphone use for a period longer than intended Persistent desire and/or unsuccessful attempts to quit or reduce smartphone use Excessive time spent on using or quitting the smartphone use Continued excessive smartphone use despite knowledge of having a persistent or recurrent physical or psychological problem resulting from smartphone overuse
**Criteria B**	Functional impairment: two (or more) of the following symptoms have been present
	Excessive smartphone use resulting in persistent or recurrent physical or psychological problem Smartphone use in a physically hazardous situation (e.g., smartphone use while driving, or crossing the street), or having other negative impacts on daily life Smartphone use resulting in impairment of social relationships, school achievement, or job performance Excessive smartphone use causes significant subjective distress, or is time-consuming
**Criteria C**	Exclusion criteria
	The smartphone addictive behavior is not better accounted for by obsessive–compulsive disorder or by bipolar I disorder.

Demographic factors and smartphone use characteristics were compared between the addicted and non-addicted groups (Table 5). The average daily use time of smartphone among addicted group was significantly longer than that among non-addicted group. However, the results did not reveal any significant difference in age, gender, duration of owning a smartphone, or the proportion of self-reported frequent users between the two groups.

**Table 5:** Comparison between the smartphone addicted and non-addicted groups according to the proposed diagnostic criteria for smartphone addiction.

	Addict group (N = 65)	Non-addict group (N = 216)	p-value
Age, year (mean (SD))	20.7 (1.4)	20.9 (1.6)	0.301
Gender, Male/Female	53/12	180/36	0.736
Frequent users	9	45	0.210
Non-frequent users	56	171	
Time spent on smartphone of non-frequent users, hours per week (mean (SD))	26.3 (17.3)	21.4 (14.2)	0.035
Duration of owning a smartphone, months (mean (SD))	30.6 (17.7)	27.4 (19.3)	0.224

Frequent users: participants thought that their smartphone use pattern was too frequent to assess the total duration; SD: Standard deviation.

## Discussion

The diagnostic criteria for smartphone addiction proposed in this study were based on the population with the current largest sample size and the diagnoses were validated by psychiatric interviews. Our findings indicated that smartphone addiction has overlapping features with substance-related or behavioral addictive disorders, but the unique properties of smartphones, i.e. its excellent accessibility and multiple Internet-based applications, contributed to its unique but prevalent addictive behaviors.

We proposed that a strict definition for smartphone-related functional impairment, requiring two or more functional impairment criteria influenced by smartphone use. This definition is different from the diagnostic criteria for functional impairment in DSM-5. Compared to computer-based Internet addiction, the portability of smartphones dampens the severity of functional impairment associated with smartphone addiction, but instead influences multiple domains of an individual’s daily life. In addition, because smartphone addiction is a heterogeneous and multi-faceted condition [26], we should evaluate the heterogeneity of its functional impairment from different angles.

The symptom criteria in this study provided evidence that smartphone addiction has similar psychopathology with the traditional substance use disorders. The core symptoms of substance use disorders are “impaired control”, which consist of four criteria: (1) use larger amounts/longer, (2) repeated attempts to quit/control use, (3) much time spent using, and (4) craving [7]. The first three criteria correspond to our criteria A-3, A-4, and A-5. In addition, our criterion A1 “recurrent failure to resist the impulse of using the smartphone” is regarded as a central component of behavior addiction [27]. Unlike substance use disorder, however, the “craving” symptom was not included in our proposed criteria. Since substance craving was only presented during a non-use period, the lack of craving in our proposed criteria implied that the smartphone has been deeply relied on in current lifestyle, so that a non-use period becomes really limited.

The excellent discriminating ability of withdrawal criteria provided evidence that withdrawal is an actual phenomenon in the manifestation of behavioral addiction syndromes, which is still under debate, even though DSM-5 has listed withdrawal as a criterion of gambling or Internet gaming disorders [13]. Our proposed withdrawal criterion A2 included mood states (e.g. dysphoria, anxiety) and active symptoms (e.g. irritability), matching the withdrawal criterion of Internet gaming disorders in an international consensus [13]. In the structured interview, we used probing questions to clarify the emotional experience few hours or two to three days after stopping using smartphones. The emotional response after a relatively long period without smartphone use (e.g. more than two weeks) could be a manifestation of craving response rather than withdrawal symptoms [11].

Tolerance is a central component of substance-related physiological dependence, but is excluded from our final diagnostic criteria because of the low diagnostic accuracy. However, the relatively high proportion (27%) among the participants was identified to have tolerance. We believe that the exclusion of tolerance just indicated that the presence of tolerance did not contribute to the diagnosis. It is conceivable that smartphone users exchange more and more information from the beginning of smartphone use. Compared with online games, individuals can easily achieve the ceiling effect of the satisfaction with smartphone use. Considering the fact that smartphone use could be essential in current lifestyle, the increasing use of smartphone, a predominant manifestation of tolerance, may not be pathological. Therefore, it is reasonable not to regard tolerance as fundamental for smartphone addiction. Furthermore, the specific feature of frequent and short-period smartphone use made the tolerance difficult to assess. Our previous study showed that tolerance had the lowest inter-rater reliability among all twelve symptom criteria [14]. Even though the psychiatrists used the App-generated parameters to define the tolerance by one-month longitudinal follow up instead of cross sectional self-reports, it remained very difficult to use tolerance criteria to facilitate the diagnosis of smartphone addiction [25].

The addicted group spent more time on smartphone than the non-addicted group. Even there is an underestimation of the smartphone use, the self-reported time spent on smartphone is moderate correlated to the App-recorded use time [14]. These results support the assertion that the diagnostic criteria proposed in this study can discriminate between those individuals with different severities of smartphone use. There is no significant difference between the addicted and non-addicted group about how long the individuals own the smartphone. This result suggested that the addiction does not depend on the duration of smartphone exposure. However, a comprehensive list of risk factors should be investigated in further studies.

As developing the first diagnostic criteria for smartphone addiction, the provisional gold standard, namely CGI from psychiatrists, was based on expert opinion rather than objective assessment. Although our previous studies showed good inter-rater reliability and test-retest reliability in the diagnostic interview, further research using brain image as the gold standard is required to validate behavioral and neurobiological similarities with recognized addictive behaviors. For example, two major brain regions in addiction research, anterior cingulate cortex and striatum, were applied to interpret the psychobiological model of Internet addiction: low inhibitory control from anterior cingulate cortex, over the strong dopaminergic bursts from striatum [28] represented an essential part of the biology of Internet addiction. This mechanism of Internet addiction also involved the core symptoms “impaired control” in smartphone addiction and shared the same psychobiological model of substance related disorders [29].

Several methodological limitations should be noted when interpreting our findings. First, the participants consisted of male-predominant college students, which might limit the generalization of the findings. Second, the high proportion of smartphone addiction by psychiatrists’ clinical global impression can lead to over-diagnosis. As a newly developed addictive behavior, we had applied a higher threshold of functional impairment to minimize the risk of over-diagnosis. Third, the diagnosis of smartphone addiction was determined solely on the basis of the participants’ responses to the structured diagnostic interview. Despite we selected the six-criterion model with optimal cutoff point by statistical methods, the diagnostic accuracies of the six criteria in the range of 70.1%-76.5% were close to the diagnostic accuracies of the other excluded six criteria in the range of 67.6%-69.9%. In multiple regression analysis, all twelve symptom criteria demonstrated statistical significance with the p-value less than 0.001 to predict the diagnosis of smartphone addiction in the regression model (S1 Table: Logistic regression model of each criterion (Criterion A1 to A12) on clinical general impression). These results of both diagnostic accuracy and regression analysis might indicate that the symptom criteria are insufficient for smartphone diagnosis. More supplementary information or App-recorded data may contribute to the confirmation of the symptoms and functional impairment criteria. Furthermore, for a continuous gold standard, several spectrum approaches such as factor analysis are powerful to detect the interaction between different criteria and to validate diagnostic tool, which is not suitable for a dichotomous gold standard (such as CGI in our case). It will be necessary to extend spectrum approaches to dichotomous dependent variable in further studies. Finally, the functional impairment should be influenced by smartphone use, which can be confounded by other psychiatric disorder such as depression. A longitudinal study should be conducted to investigate the causal relationship between functional impairment and smartphone use, as well as the time course of the symptom development.

In conclusion, this study established the diagnostic criteria of smartphone addiction. The core symptoms focus on impaired control and are paralleled with substance related and addictive disorders. Considering the unique feature of smartphone use, the functional impairment secondary to smartphone use should be assessed in multiple domains.

## Supporting Information

S1 Table Logistic regression model of each criterion (Criterion A1 to A12) on clinical general impression. (DOCX)
