# Supplementary material for: Proposed Diagnostic Criteria for Smartphone Addiction
Source: PLoS One. 2016 Nov 15;11(11):e0163010. doi: 10.1371/journal.pone.0163010 (PMC5112893; doi:10.1371/journal.pone.0163010)
Supplement: S1 Table — (DOCX) [file pone.0163010.s001.docx]

Supplementary Table. Logistic regression model of each criterion (Criterion A1 to A12) on clinical general impression

|  | Odds Ratio | 95% CI | |
| --- | --- | --- | --- |
| A1 | 4.027 | 2.348 | 6.908 |
| A2 | 7.033 | 4.021 | 12.300 |
| A3 | 3.117 | 1.806 | 5.379 |
| A4 | 4.079 | 2.308 | 7.208 |
| A5 | 5.091 | 2.991 | 8.665 |
| A6 | 4.257 | 2.399 | 7.555 |
| A7 | 10.284 | 5.784 | 18.285 |
| A8 | 4.433 | 2.624 | 7.490 |
| A9 | 8.091 | 4.564 | 14.344 |
| A10 | 3.978 | 2.278 | 6.948 |
| A11 | 4.649 | 2.145 | 10.074 |
| A12 | 5.398 | 2.265 | 12.864 |

All p-values are lower than 0.001. CI: Confidence Interval
